# Localization of Parathyroid Disease in Reoperative Patients with Primary Hyperparathyroidism

**DOI:** 10.1155/2020/9649564

**Published:** 2020-01-25

**Authors:** Aaroh M. Parikh, Raymon H. Grogan, Fanny E. Morón

**Affiliations:** ^1^School of Medicine, Baylor College of Medicine, Houston, TX 77030, USA; ^2^Department of Internal Medicine, Santa Clara Valley Medical Center, San Jose, CA 95128, USA; ^3^Michael E. DeBakey Department of Surgery, Baylor College of Medicine, Houston, TX 77030, USA; ^4^Department of Radiology, Baylor College of Medicine, Houston, TX 77030, USA

## Abstract

The localization of persistent or recurrent disease in reoperative patients with primary hyperparathyroidism presents challenges for radiologists and surgeons alike. In this article, we summarize the relevant imaging modalities, compare their accuracy in identifying reoperative disease, and outline their advantages and disadvantages. Accurate localization by preoperative imaging is a predictor of operative success, whereas negative or discordant preoperative imaging is a risk factor for operative failure. Ultrasound is a common first-line modality because it is inexpensive, accessible, and radiation-free. However, it is highly operator-dependent and less accurate in the reoperative setting than in the primary setting. Sestamibi scintigraphy is superior to ultrasound in localizing reoperative disease but requires radiation, prolonged imaging times, and reader experience for accurate interpretation. Like ultrasound, sestamibi scintigraphy is less accurate in the reoperative setting because reoperative patients can exhibit distorted anatomy, altered perfusion of remaining glands, and interference of radiotracer uptake. Meanwhile, four-dimensional computed tomography (4DCT) is superior to ultrasound and sestamibi scintigraphy in localizing reoperative disease but requires the use of radiation and intravenous contrast. Both 4DCT and magnetic resonance imaging (MRI) do not significantly differ in accuracy between unexplored and reoperative patients. However, MRI is more costly, inaccessible, and time-consuming than 4DCT and is inappropriate as a first-line modality. Hybrid imaging with positron emission tomography and computed tomography (PET/CT) may be a promising second-line modality in the reoperative setting, particularly when first-line modalities are discordant or inconclusive. Lastly, selective venous sampling should be reserved for challenging cases in which noninvasive modalities are negative or discordant. In the challenging population of reoperative patients with PHPT, a multimodality approach that utilizes the expertise of high-volume centers can accurately localize persistent or recurrent disease and enable curative parathyroidectomy.

## 1. Introduction

Reoperation in patients with primary hyperparathyroidism (PHPT) presents unique challenges for both radiologists and surgeons. Although the vast majority of patients with PHPT undergo curative initial exploration, between 2 and 10% of patients develop persistent or recurrent disease after initial surgery [1–9]. Persistent PHPT (pPHPT) is defined as elevated serum calcium and parathyroid hormone (PTH) levels within 6 months of initial surgery. Recurrent PHPT (rPHPT) is defined as elevated serum calcium and PTH levels more than 6 months after successful surgery. Accurate preoperative localization has enabled minimally invasive parathyroidectomy to be as successful as traditional bilateral approaches [2, 4, 10]. In the reoperative setting, repeat surgery is not recommended unless the lesion can be well-localized.

In this article, we review the localization of parathyroid disease in the reoperative setting. We summarize the relevant imaging modalities, compare their accuracy in identifying reoperative disease, and outline their advantages and disadvantages.

## 2. Common Causes of Initial Postsurgical Failure

**Figure 1:**
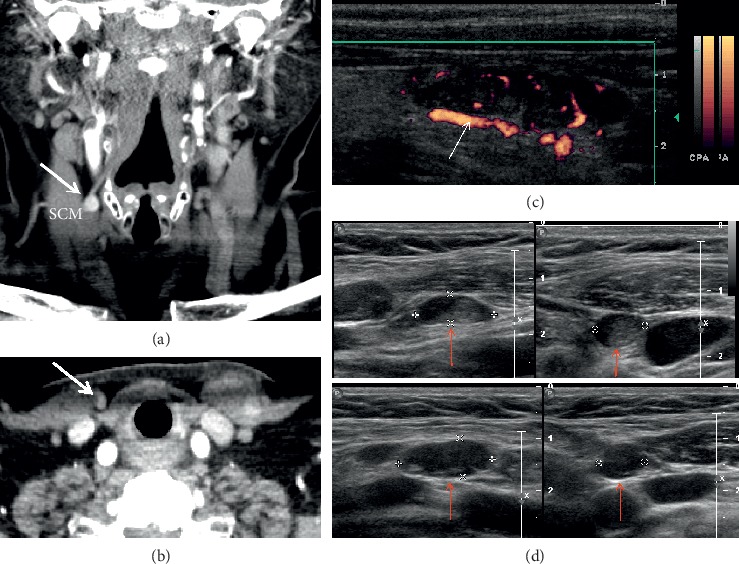
Autotransplanted parathyroid gland and parathyromatosis. A 70-year-old female presents with hyperparathyroidism after remote thyroidectomy. Coronal (a) and axial (b) four-dimensional computed tomography (4DCT) shows a hyperenhancing parathyroid gland (thick arrows) superficial to the right sternocleidomastoid muscle (SCM). Power Doppler ultrasound (c) of the autotransplanted hypoechoic nodule shows a polar feeding vessel (thin arrow) and peripheral rim of vascularity. Additional well-circumscribed mostly hypoechoic nodules consistent with multiple implants of parathyroid tissue (d) (red arrows) are also visualized.

**Figure 2:**
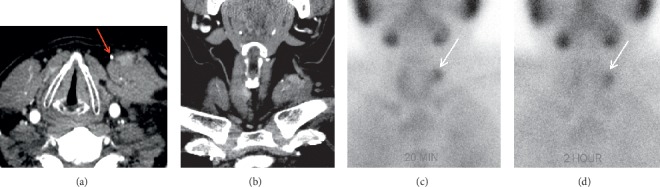
Hyperfunctioning autotransplanted parathyroid remnant. A 50-year-old male with multiple endocrine neoplasia type 1 (MEN1) presents with recurrent disease after prior parathyroidectomy. Axial (a) and coronal (b) four-dimensional computed tomography (4DCT) images show an irregular enhancing focus (white arrows) superficial to the left SCM muscle, lateral to a surgical clip (red arrow). Planar immediate (c) (20 minutes) and delayed (d) (two hours) single-photon emission computed tomography (SPECT) images demonstrate focal radiotracer accumulation (white arrows) in the left lateral neck that persists on delayed images and confirms the diagnosis of the hyperfunctioning parathyroid gland.

The most common causes of failed initial parathyroidectomy include missed orthotopic adenoma, ectopic adenoma, and multigland disease (encompassing both multiple adenomas and multigland hyperplasia), whereas less common causes include parathyroid carcinoma, regrowth of a partially resected gland, and parathyromatosis from inadvertent seeding of parathyroid cells during prior exploration [1, 11–17]. While some studies report that multigland disease accounts for more than half of all reoperative cases [3, 11], most studies attribute the majority of reoperative cases (as high as 79%) to the failed detection of a single adenoma [1, 13, 15–19]. Although ectopic adenomas may be up to four times as common in the reoperative setting than in the primary setting [11, 18, 20, 21], the majority of missed single adenomas are orthotopic [4, 14, 19, 22, 23]. Multiple endocrine neoplasia type 1 (MEN1) specifically accounts for between 8.5 and 10.3% of all reoperative cases, causing both pPHPT and rPHPT [11, 15]. More than one-third of patients with MEN1 are undiagnosed upon initial operation, and approximately half develop persistent disease after initial operation [24]. Rarer causes of initial failure include parathyromatosis (Figure 1), parathyroid carcinoma, and adenoma arising from autotransplanted parathyroid glands following surgical exploration (Figures 1 and 2), each accounting for ≤3% of reoperative cases [12, 19, 25, 26]. The development of a second adenoma in a previously normal gland is rare except for in patients with prior neck radiation [27].

## 3. Role of Preoperative Imaging

Imaging is not diagnostic of PHPT but is used to localize disease prior to surgical exploration. Indications for preoperative imaging include determining candidacy for minimally invasive parathyroidectomy, assessing for ectopic glands, assessing for concurrent thyroid neoplasia, and evaluating persistent or recurrent disease after prior parathyroidectomy [28, 29]. Accurate preoperative localization identifies ectopic glands, decreases operating time, decreases the likelihood of surgical complications, and improves the success rate of parathyroidectomy [11, 30, 31]. Meanwhile, negative or discordant preoperative imaging is a risk factor for persistent disease after surgical exploration [3, 32], with initial surgical failure rates higher than those for well-localized glands [28, 32]. Rates of inconclusive first-line imaging have been reported as high as 63% in the reoperative setting [12]. Particularly in this challenging population, the expertise of high-volume centers may increase the yield from preoperative imaging modalities [33].

## 4. Surgical Reexploration

Before proceeding to repeat surgical exploration, patients with pPHPT or rPHPT should have their diagnosis biochemically reconfirmed and undergo additional (repeat) preoperative imaging localization, alongside a full review of prior imaging and operative findings [34]. In addition, patients need to be reassessed for symptoms associated with PHPT to ensure that they have an indication for reoperation.

Reoperation can be technically challenging due to scarring and obliteration of tissue planes, obscuring of normal anatomy of recurrent and superior laryngeal nerves, and encasement of abnormal parathyroid glands in scar tissue following initial surgery [4, 29]. Due to the difficulties of localizing and excising abnormal glands in the reoperative setting, repeat parathyroidectomy used to have operative success rates between 82 and 90%, lower than the analogous rates for initial exploration [35, 36]. Today, however, surgical cure rates in the reoperative setting are reported between 86 and 100%, with a meta-analysis by Singh Ospina and colleagues reporting a cure rate of 98% with bilateral neck exploration and 97% with minimally invasive parathyroidectomy [4, 12, 13, 37–41]. Common complications after reoperation include injury to the recurrent laryngeal nerve and permanent hypoparathyroidism, the reported frequencies of which vary widely from 0 to 15% for each [4, 12, 22, 40–42]. Given the challenges that accompany reoperative parathyroidectomy, it is crucial to seek the expertise of high-volume and experienced parathyroid surgeons when assessing a patient with pPHPT or rPHPT [7, 34].

## 5. Ultrasound

Ultrasound is a common first-line modality used to preoperatively localize parathyroid adenomas. Ultrasound localization requires examination of the anterior cervical region using a high-frequency linear transducer. Parathyroid adenomas appear round or ovoid, well-circumscribed, homogeneous, and hypoechoic relative to thyroid tissue (Figure 1). Doppler interrogation often identifies a polar vessel or peripheral rim of vascularity surrounding the adenomatous gland (Figure 1). Ultrasound also guides fine-needle aspiration of possible parathyroid lesions, albeit accompanied by the risk of causing fibrosis of the adenoma and surrounding tissues. Meta-analysis of preoperative ultrasound has determined a pooled sensitivity of 76% and a pooled positive predictive value (PPV) of 93% for localizing abnormal parathyroid glands in *de novo* patients with PHPT [43]. However, the accuracy of ultrasound decreases in the reoperative setting. In patients with pPHPT or rPHPT undergoing reoperative parathyroidectomy, the sensitivity of ultrasound for localizing abnormal glands ranges between 54 and 68% [4, 15, 23, 44]. Although there are studies that report analogous sensitivities between 20 and 40%, such studies include very few reoperative patients [45] or use samples that specifically required a second-line modality for accurate localization [37]. Furthermore, ultrasound possesses significantly lower sensitivity (often reported near 40%) in patients with multigland disease, a population that frequently requires reoperation [2, 37, 39, 45]. A systematic review of 20,225 cases of PHPT determined that the sensitivity of preoperative ultrasound is 79% for localizing solitary adenomas but decreases to 34.9% for localizing multigland hyperplasia and 16.2% for localizing double adenomas [2].

Ultrasound is an attractive first-line modality because it is inexpensive, accessible, and radiation-free. It also enables fine-needle aspiration of suspected parathyroid lesions and evaluation of concurrent thyroid pathology. However, ultrasound is subject to several limitations. It is highly operator-dependent and is often insensitive to multigland parathyroid disease [2, 28, 37, 39, 45–47]. Ultrasound frequently fails to localize adenomatous glands in obese patients or in sonographically inaccessible locations (e.g., mediastinal, tracheoesophageal groove, retroesophageal, and retroclavicular) [21, 28, 46, 47]. Ultrasound is well-suited as a first-line modality in both primary and reoperative settings, particularly at high-volume institutions where radiologists and surgeons have familiarity and confidence with the modality. Nevertheless, patients with pPHPT or rPHPT should still undergo additional imaging studies for more accurate preoperative localization.

## 6. Sestamibi Scintigraphy

**Figure 3:**
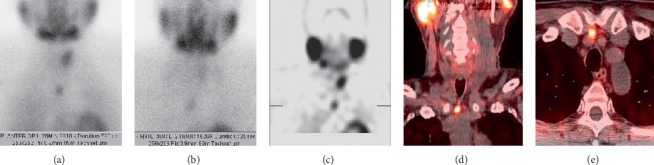
Ectopic mediastinal adenoma. A 56-year-old female presents with persistent hyperparathyroidism after prior four-gland parathyroidectomy and right thyroid lobectomy. Planar sestamibi scintigraphy at 20 minutes (a) and two hours (b) and coronal single-photon emission computed tomography (SPECT) (c) show a hypermetabolic mediastinal gland. Coronal (d) and axial (e) hybrid SPECT and computed tomography (SPECT/CT) confirm an ectopic and adenomatous fifth gland in the anterior mediastinum.

**Figure 4:**
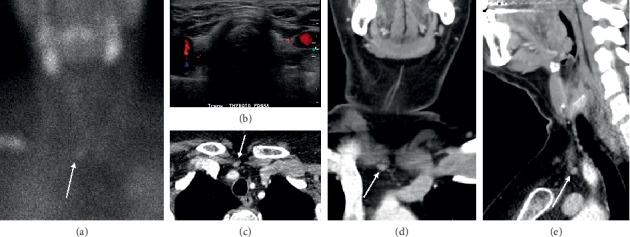
Inconclusive sestamibi scintigraphy versus ultrasound with successful localization by four-dimensional computed tomography (4DCT). A 49-year-old female with multiple endocrine neoplasia type 1 (MEN1) and remote prior three-gland parathyroidectomy presents with recurrent hyperparathyroidism. Planar delayed (two hours) sestamibi scintigraphy (a) shows a questionable-inconclusive enlarged right hypermetabolic parathyroid gland in the superior mediastinum (white arrow). Ultrasound with Doppler interrogation (b) is negative for recurrence. Axial (c), coronal (d), and sagittal (e) arterial phase 4DCT was performed to clarify discordant first-line findings and confirm a small hyperenhancing and ectopic right intrathymic parathyroid adenoma (white arrows).

**Figure 5:**
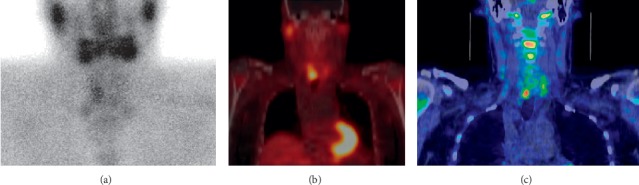
Persistent hyperparathyroidism. Preoperative ^123^I/^99m^Tc subtraction scintigraphy (a), ^99m^Tc sestamibi hybrid single-photon emission computed tomography and computed tomography (SPECT/CT) (b), and ^11^C-methionine hybrid positron emission tomography and computed tomography (PET/CT) (c) correctly localized the pathologic parathyroid gland (bottom right). Histology demonstrated parathyroid glandular hyperplasia (figure reused with permission).

Sestamibi scintigraphy is frequently used to identify abnormal parathyroid glands. The most common protocol consists of dual-phase single-isotope scintigraphy using the ^99m^Tc sestamibi radiotracer. The radiotracer concentrates in the mitochondria of metabolically active tissues, such as the mitochondria-rich oxyphil cells of hyperfunctioning parathyroid glands [48]. The early phase (15 minutes after injection) demonstrates radiotracer uptake in both thyroid and parathyroid glands. The delayed phase (120 minutes after injection) demonstrates washout of the radiotracer from normal tissue but retention of the radiotracer in hyperfunctioning parathyroid glands (Figures 2 and 3). Meta-analysis of dual-phase ^99m^Tc sestamibi scintigraphy has demonstrated a pooled sensitivity of 63% and a pooled PPV of 90% for localizing abnormal glands in patients with PHPT [49]. However, like that of ultrasound, the accuracy of sestamibi scintigraphy decreases in the reoperative setting (Figure 4). In patients with pPHPT or rPHPT undergoing reoperative parathyroidectomy, the sensitivity of ^99m^Tc sestamibi scintigraphy for localizing parathyroid adenomas ranges from 53 to 74% [4, 23, 50]. Single-photon emission computed tomography (SPECT) and hybrid imaging with both SPECT and computed tomography (SPECT/CT) offer additional anatomic visualization that aids in surgical planning (Figure 3). However, the addition of SPECT and SPECT/CT does not markedly change the sensitivity of the technique (between 69 and 74%) [44, 51]. A small number of studies have investigated the use of ^123^I/^99m^Tc subtraction scintigraphy in the reoperative setting with sensitivities reported as high as 81%, leading some to advocate for its use over traditional single-tracer scintigraphy (Figure 5) [15, 40, 50, 52]. Dual-phase ^99m^Tc sestamibi scintigraphy is significantly less accurate at localizing multigland disease as compared to single-gland disease, with sensitivities reported between 23 and 45% [2, 37, 39, 45, 50]. A 2005 meta-analysis reported that the sensitivity of preoperative sestamibi scintigraphy decreases from 88.4% for localizing solitary adenomas to 44.5% for localizing multigland hyperplasia and 30.0% for localizing double adenomas [2].

Sestamibi scintigraphy is an operator-independent modality that offers both functional and anatomic information when supplemented by computed tomography as in SPECT/CT. Although sestamibi scintigraphy is a commonly used first-line modality in both primary and reoperative settings, it possesses clear limitations. Sestamibi scintigraphy requires relatively long imaging times as well as radiation exposure. It also requires experience to interpret accurately, underscoring the importance of seeking a high-volume radiologist. It is quite limited in both its anatomic resolution and its ability to localize small parathyroid glands or multigland disease (in which glands tend to be only mildly enlarged) [14, 28, 53–55]. There are several factors that may explain why the accuracy of preoperative sestamibi scintigraphy decreases in the setting of persistent or recurrent disease. Dual-phase sestamibi techniques often fail to localize hyperplastic glands (which are a common cause for reoperation) because they do not retain the radiotracer during the late phase [56–59]. Patients having undergone prior exploration may exhibit distorted anatomy, altered perfusion of remaining glands, and interference of ^99m^Tc uptake, all of which decrease the accuracy of sestamibi scintigraphy in the reoperative setting [60–62]. Despite these drawbacks, sestamibi scintigraphy still possesses a higher sensitivity than ultrasound for localizing reoperative disease, ectopic disease, and multigland disease [2, 4, 21, 37, 46]. Consequently, sestamibi scintigraphy remains an adequate first-line modality for preoperatively localizing reoperative disease.

## 7. Four-Dimensional Computed Tomography

**Figure 6:**
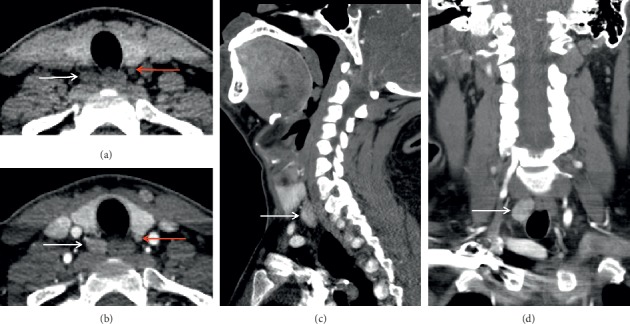
Multigland disease on four-dimensional computed tomography (4DCT). A 44-year-old female presents with recurrent hyperparathyroidism after prior right inferior parathyroidectomy. Axial precontrast (a) and postcontrast (b) early arterial phases show a moderately enhancing overly descended right superior parathyroid adenoma (white arrows) and a small hyperenhancing orthotopic left inferior parathyroid adenoma (red arrows). Coronal (c) and sagittal (d) views similarly demonstrate the overly descended right superior parathyroid adenoma (white arrows).

**Figure 7:**
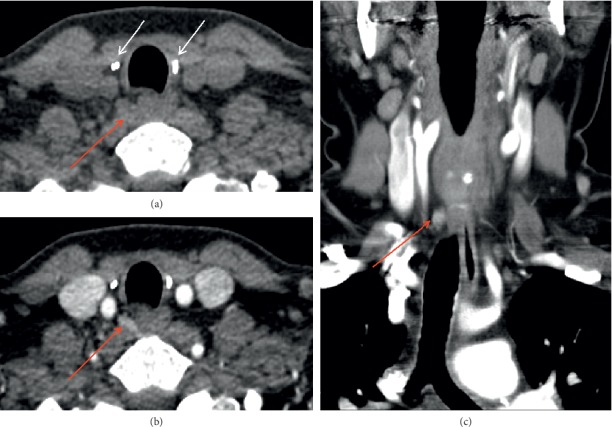
Ectopic parathyroid adenoma on four-dimensional computed tomography (4DCT). A patient presents with recurrent hyperparathyroidism after prior total thyroidectomy with benign pathology and prior bilateral inferior parathyroidectomy. Axial precontrast 4DCT (a) shows ectopic adenoma (red arrow) in the right para-retroesophageal region almost at the prevertebral fascia, deep to right surgical clips (white arrows). The ectopic adenoma is hyperenhancing on axial postcontrast (b) and coronal (c) early arterial phases (red arrows).

The advent of four-dimensional computed tomography (4DCT) has facilitated accurate preoperative localization of abnormal parathyroid glands. 4DCT consists of multiphase computed tomography—often using noncontrast, arterial, and delayed (venous) phases—to detect changes in enhancement over time. Parathyroid adenomas exhibit low attenuation (compared to normal thyroid tissue) on noncontrast images, peak enhancement during the arterial phase, and washout of contrast in the delayed phase (Figures 6 and 7). As a first-line study in *de novo* or uncomplicated patients with PHPT, 4DCT has sensitivity to localization between 62 and 92% and a PPV between 88 and 94% [28]. In the reoperative setting, 4DCT has sensitivity for localization as high as 93% and sensitivity for lateralization as high as 97% [28, 37, 60, 63–65]. However, this modality exhibits variable accuracy in patients with multigland disease (Figure 6). Although 4DCT can correctly predict multigland disease in 80 to 90% of patients with surgically proven multigland disease (in studies that pooled both *de novo* and reoperative patients) [60, 65], its sensitivity for accurate localization of multigland disease ranges from 43 to 69% [37, 45, 66, 67]. Between 57 and 75% of lesions missed by 4DCT in the reoperative setting constitute multigland disease [64, 68]. In addition to comprising a substantial proportion of reoperative patients, patients with multigland disease are also more likely to have milder hypercalcemia and smaller lesions than patients with single-gland disease [67, 69]. 4DCT is significantly more accurate than sestamibi scintigraphy at lateralizing parathyroid lesions in patients with mild hypercalcemia (<10.8 mg/dL) and low gland weights (<0.5 g) [70]. Another risk factor for requiring reoperation is nonlocalizing, inconclusive, or discordant first-line imaging studies (Figure 4). When used as a second-line modality in patients with nonlocalizing, inconclusive, or discordant prior imaging, 4DCT has sensitivity for localization between 67 and 89% and a PPV between 65 and 87% (Figure 4) [28, 45, 71]. In a study of reoperative patients specifically with negative ultrasound and sestamibi scintigraphy, 4DCT had sensitivity for localization of 50% and a PPV of 100% [72].

4DCT has many strengths that make it well-suited for the reoperative setting. Due to its very high spatial resolution, 4DCT delineates important anatomic landmarks and structures surrounding the diseased gland(s), thereby providing critical information that can guide surgical reexploration. It requires short imaging times and can capably localize ectopic adenomas (Figure 7) [21, 46]. 4DCT also has important disadvantages. It confers ionizing radiation, uses iodinated contrast, and requires radiologist experience to accurately interpret the modality. 4DCT has limited accuracy in patients with multigland disease, small glands, or concomitant thyroid pathology [73]. Nevertheless, 4DCT has emerged as a useful and sensitive modality in the setting of reoperation. Unlike ultrasound and sestamibi scintigraphy, 4DCT does not significantly differ in accuracy between unexplored patients and reoperative patients [63]. The modality is also superior to ultrasound and sestamibi scintigraphy in the preoperative localization of persistent or recurrent disease [37, 64, 71, 74, 75]. In addition, 4DCT accurately identifies parathyroid disease in challenging reoperative patients, such as those with negative first-line imaging or multigland disease [28, 37, 45, 66, 67, 72]. Finally, because it can differentiate unilateral and bilateral disease in up to 96% of reoperative patients, 4DCT can enable targeted parathyroidectomies in difficult reoperative cases [37, 39, 76]. Thus, 4DCT possesses several attributes that make it an accurate and informative modality for the preoperative localization of persistent or recurrent disease.

## 8. Magnetic Resonance Imaging

**Figure 8:**
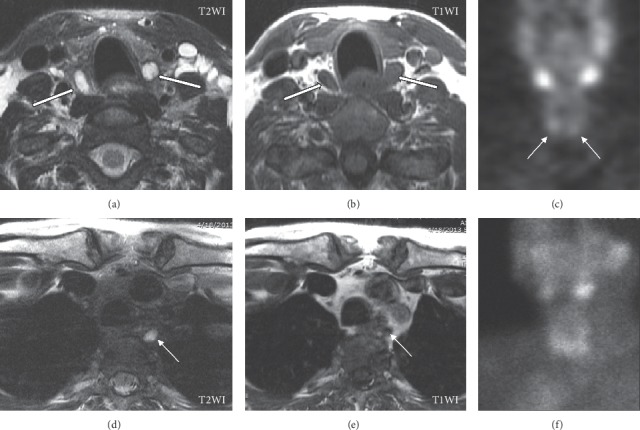
Parathyroid adenomas on magnetic resonance imaging (MRI). A 67-year-old female presents with persistent hyperparathyroidism after initial bilateral inferior parathyroidectomy. Unenhanced MRI (due to allergy to iodine- and gadolinium-based contrast materials) shows well-circumscribed bilateral inferior T2WI hyperintense (a) and T1WI hypointense (b) adenomas (white arrows) posterior to the inferior thyroid lobes. Planar delayed (two hours) single-photon emission computed tomography (SPECT) (c) demonstrates focal radiotracer accumulation (white arrows) along the inferior margin of the thyroid lobes. Further review of the MRI reveals small T2WI hyperintense (d) and poorly defined T1WI hypointense (e) nodules in the left posterior mediastinum/prevertebral region corresponding to an overly descended left superior parathyroid adenoma not demonstrated on initial SPECT studies (c, f).

**Figure 9:**
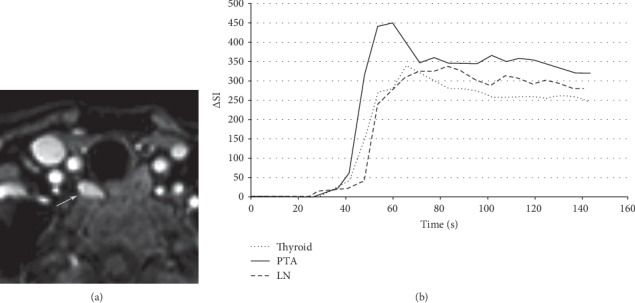
Parathyroid adenoma on dynamic 4D contrast-enhanced magnetic resonance imaging (MRI). A 68-year-old female presents with primary hyperparathyroidism. An axial arterial phase contrast-enhanced image from magnetic resonance perfusion demonstrates a parathyroid adenoma (arrow) in the right tracheoesophageal groove. Concentration-time curve analysis from regions of interest placed over the parathyroid adenoma (arrow), thyroid gland, and a jugulodigastric lymph node shows significantly faster time-to-peak (TTP) and higher wash-in and washout values of the parathyroid adenoma compared to the thyroid gland and cervical lymph node. Parathyroid adenoma: TTP, 37 seconds; wash-in, 7.8; washout, 0.58. Thyroid: TTP, 42 seconds; wash-in, 5.4; washout, 0.46. Lymph node: TTP, 60 seconds; wash-in, 4.8; washout, 0.29. ΔSI indicates the change in signal intensity. Wash-in is the initial upslope of the concentration-time curve (slope from the end of the baseline to the peak of the curve). Washout is the downslope of the concentration-time curve (negative slope from the peak to the last acquisition time point) (figure reused with permission).

Magnetic resonance imaging (MRI) is occasionally used as a second-line modality to identify lesions that have been otherwise poorly or inconclusively localized by prior studies. Conventional MRI protocols for preoperatively localizing parathyroid lesions include small field-of-view precontrast axial T1- and T2-weighted sequences and postcontrast T1-weighted images with fat saturation. Parathyroid adenomas appear isointense to the muscle on T1-weighted images, hyperintense on T2-weighted images, and strongly and rapidly enhancing on postcontrast fat-saturated T1-weighted images (Figures 8 and 9) [44]. Such protocols have demonstrated sensitivities as high as 91% for localizing abnormal glands in patients with PHPT [46]. The addition of MRI to the combination of ultrasound and sestamibi scintigraphy significantly increases the sensitivity for localization from 75% to 92% [44]. In the reoperative setting, meanwhile, multiple investigations have reported the sensitivity of conventional MRI for localizing parathyroid adenomas to be 82% [44, 77]. A small number of studies have investigated the role of dynamic MRI. Dynamic 4D contrast-enhanced (DCE) MRI is a multiphase (4-phase) contrast-enhanced high spatial and temporal resolution T1-weighted sequence with fat suppression. Like 4DCT, dynamic MRI makes use of the hypervascular behavior of parathyroid adenomas. DCE provides quantitative perfusion parameters that enable differentiation between parathyroid adenomas, lymph nodes, and thyroid tissue. On dynamic MRI, parathyroid adenomas exhibit significantly faster arterial enhancement and higher wash-in and higher washout compared to lymph nodes and thyroid tissue (Figure 9) [78]. In unselected patients with PHPT, dynamic MRI has a reported sensitivity of 91% for detecting parathyroid adenomas [78]. In reoperative patients, dynamic MRI has a sensitivity of 90% for localizing adenomas; although this sensitivity is higher than that of conventional MRI in the reoperative setting (82%), this difference was not statistically significant [44]. The addition of dynamic magnetic resonance angiography (MRA) has also been investigated as a localizing modality for parathyroid adenomas. MRA constitutes a contrast-enhanced 3D angiographic acquisition. Parathyroid adenomas appear hyperenhancing during the early arterial phase, while thyroid tissue enhances during subsequent phases. In a study of 30 patients with hyperparathyroidism and prior neck surgery, MRI with dynamic MRA had sensitivity for localization of 93% [53].

Unlike sestamibi scintigraphy and 4DCT, MRI does not confer ionizing radiation, nor does it have significantly lower sensitivity in patients with concomitant thyroid pathology [44]. MRI also possesses specific advantages that make it well-suited to evaluate reoperative disease. MRI does not significantly differ in sensitivity between unexplored patients and reoperative patients, as reported by Kluijfhout and colleagues in a study of 41 unexplored and 84 reoperative patients with PHPT [44]. The addition of MRI to first-line modalities significantly increases the sensitivity to adenoma localization [44]. Although this increase was shown in a sample consisting of both initial and reoperative patients, it is reasonable to conclude that MRI provides an added benefit in the reoperative setting, where the accuracy of ultrasound and sestamibi scintigraphy markedly suffer. While the use of dynamic MRI does not confer a significant benefit over conventional MRI in the reoperative setting, the addition of dynamic MRA might, as it enables the detection of smaller adenomas than conventional MRI alone [53]. Yet, these strengths must be weighed against both the drawbacks of the modality and the limitations in evidence supporting it. MRI is more costly, inaccessible, and time-consuming than 4DCT and would be inappropriate as a first-line imaging modality. Only a small handful of investigations with narrow samples support the use of dynamic MRI or dynamic MRA in preoperatively localizing persistent or recurrent disease. Despite the high sensitivities of MRI for localizing reoperative lesions, further investigation is necessary before conclusively establishing the role of MRI in patients with persistent or recurrent disease.

## 9. Positron Emission Tomography and Hybrid PET/CT

**Figure 10:**
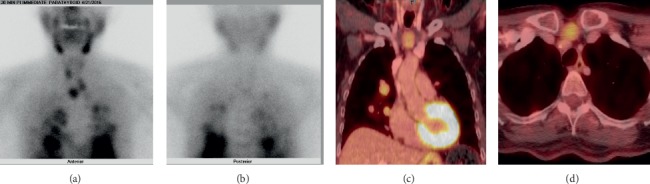
Parathyroid carcinoma. A 68-year-old female presents with recurrent hyperparathyroidism due to recurrent parathyroid carcinoma. Planar anterior (a) and posterior (b) sestamibi scintigraphy at 20 minutes and ^18^F-fluorodeoxyglucose (^18^F-FDG) hybrid positron emission tomography and computed tomography (PET/CT) show multiple marked FDG-avid lesions (c, d) attributable to both local recurrence and multiple local and lung metastases.

In positron emission tomography (PET), patients undergo injection of a radiotracer that demonstrates avidity for metabolically active tissues. Hyperfunctioning tissues such as parathyroid adenomas appear as focal areas of radiotracer uptake. Compared to the aforementioned modalities, PET and hybrid imaging with both PET and low-dose computed tomography (PET/CT) are relatively new localization techniques. In a pooled sample of both unexplored and reoperative patients, meta-analysis of ^11^C-methionine (^11^C-MET) PET showed a pooled sensitivity for localizing abnormal parathyroid glands of 77% and a pooled PPV of 98% [79]. Specifically in reoperative patients with PHPT, ^11^C-MET PET has a sensitivity between 75 and 88% [80–84]. Analogously, ^18^F-fluorodeoxyglucose (^18^F-FDG) PET has a sensitivity of 62% in the reoperative setting [85]. Meanwhile, hybrid imaging with PET/CT offers high-resolution anatomic information in addition to the functional information provided by PET alone. ^11^C-MET PET/CT has a sensitivity of 61% in reoperative patients, though this figure may be as low as 40% in challenging subgroups such as those with negative sestamibi scintigraphy (Figure 5) [40, 86]. However, ^18^F-fluoromethylcholine (^18^F-FCH) PET/CT demonstrates strong potential for localizing persistent or recurrent disease with sensitivities between 96 and 100% (albeit in studies with small sample sizes) [22, 87]. In patients with multigland disease, ^18^F-FCH PET/CT has a reported sensitivity of 79% and PPV of 100% [87]. Moreover, the use of both ^18^F-FDG and ^18^F-FCH in hybrid PET/CT has proven useful in evaluating parathyroid carcinoma for the extent of primary disease, metastases, and recurrence (Figure 10) [88, 89].

Preoperative localization with PET and PET/CT possesses distinct advantages. First, PET offers better spatial and temporal resolution than SPECT, thus enabling the detection of very small pathologic glands [79]. Analogously, ^18^F-FCH PET/CT has higher spatial resolution, lower radiation burden (2.8 mSv), and shorter total study time (38 minutes) than SPECT/CT (11.8 mSv, total study time 120 minutes) [90–92]. In a study of 29 patients undergoing reoperation for PHPT, ^18^F-FCH PET/CT was more sensitive than ultrasound, sestamibi scintigraphy, and 4DCT for localizing parathyroid disease; however, it is likely that patients who proceed to PET/CT have negative or discordant results from more commonly utilized modalities in the first place [22]. Moreover, ^18^F-FCH PET/CT often fails to detect hyperplastic glands and ectopic adenomas, both of which constitute common causes of reoperation [87, 93–95]. PET/CT is also an infrequently used modality and can be quite costly. Additional studies with larger samples are necessary before definitively establishing the role of PET/CT in localizing persistent or recurrent disease. Nevertheless, PET/CT may be a promising second-line modality in the reoperative setting as evidenced by its very high sensitivities, particularly when first-line imaging modalities are discordant or inconclusive.

## 10. Selective Venous Sampling

In the reoperative setting, noninvasive imaging modalities frequently fail to definitively localize disease and often demonstrate low concordance with operative findings [1, 23]. Patients with pPHPT or rPHPT as well as nonlocalizing, equivocal, or discordant noninvasive imaging studies may undergo invasive localization in the form of selective venous sampling (SVS) for PTH. SVS involves selective catheterization of neck and mediastinal veins (e.g., internal jugular veins, brachiocephalic veins, azygos vein, and vertebral veins) with PTH sampling. A PTH level in a selected vein at least twice the systemic level is considered localizing for abnormal parathyroid glands. A 2018 meta-analysis of SVS in both *de novo* (minority) and reoperative (majority) patients reported a pooled sensitivity of 74% for localizing parathyroid adenomas [96]. Among reoperative patients with inconclusive noninvasive imaging studies, the sensitivity of SVS ranges from 75 to 93% [1, 6, 41, 72]. Additionally, in reoperative patients with negative or nonlocalizing first-line imaging, the combination of 4DCT and SVS has a sensitivity of 95% and is significantly more sensitive than 4DCT alone [72]. The recent adoption of superselective venous sampling (SSVS) has increased the accuracy of invasive PTH sampling. SSVS obtains samples from smaller neck and upper chest veins (e.g., superior, middle, and inferior thyroid veins; main inferior thyroid trunk; thymic vein; superior intercostal veins; and occasionally internal mammary veins) to enable more precise localization of parathyroid pathology. In the reoperative setting, meta-analysis of SSVS has found a pooled sensitivity of 90% for localizing parathyroid adenomas [96]. Among reoperative patients with inconclusive first-line imaging, SSVS has a sensitivity of 96% and a PPV of 84%, which is significantly more accurate than routine SVS [97].

The benefits of invasive localization should be measured against its costs and limitations. Meta-analysis has shown that SVS is more sensitive than noninvasive imaging modalities at localizing adenomas in patients with pPHPT or rPHPT undergoing reoperation [96]. Yet, this higher sensitivity of SVS may be attributable to the fact that the patients enrolled in comparative studies only underwent invasive imaging because their noninvasive imaging studies were inconclusive to begin with. Localization using SVS can be inaccurate when veins drain bilaterally or in patients with altered venous anatomy and drainage patterns (as is seen in the reoperative setting). As a result, SVS may occasionally demonstrate low concordance with surgical findings in reoperative patients [98]. SVS also requires the expertise of interventional radiology and carries procedural risks that do not accompany noninvasive localization techniques. Although the combination of 4DCT and SVS and the advent of SSVS exhibit strong potential, their utility must be carefully weighed against their costs, the risks of invasive localization, and the risk of inaccurate results due to postoperative anatomic changes. As a result, SVS should be reserved for challenging reoperative cases in which noninvasive modalities are negative or discordant.

## 11. Intraoperative PTH Monitoring

Intraoperative PTH monitoring (IOPTH) is frequently utilized as a surgical adjunct to determine if all abnormal parathyroid glands have been resected. Its use has enabled minimally invasive or focused parathyroidectomy in initial explorations, as it can determine whether the remaining unexplored parathyroid glands are producing physiologic amounts of PTH. Due to the short half-life of PTH, a decrease of >50% in intact PTH from preexcision levels in a sample of peripheral blood collected 5 and 10 minutes after excision of suspected abnormal parathyroid tissue is associated with a high rate of cure. Furthermore, a decrease in IOPTH levels into the normal range provides reliable evidence that the remaining unexplored parathyroid glands are producing physiologic amounts of PTH, thus making further exploration unnecessary. Meanwhile, a persistently elevated IOPTH level may predict multigland disease in the setting of a focused exploration or may predict an ectopic adenoma in the setting of a four-gland exploration. In the reoperative setting, IOPTH has been reported to have sensitivity for predicting cure between 99 and 100% and has been shown to be a statistically significant predictor of operative success [4, 15, 23, 51]. Specifically in reoperative patients with MEN1, IOPTH has a sensitivity of 92% and accurately distinguishes patients with previously undetected multigland disease [24]. Existing literature provides mixed evidence regarding the influence of IOPTH on the rates of cure and complications [4, 15, 16]. Nevertheless, IOPTH provides critical information during the surgical exploration of challenging reoperative patients.

## 12. Radiation Dose Exposure and Associated Cancer Risks

Concerns over radiation exposure have prompted several investigations into the radiation doses conferred by preoperative imaging modalities. Sestamibi scintigraphy has an effective dose between 3.3 and 13.7 mSv, with SPECT/CT conferring more radiation than SPECT and planar scintigraphy [45, 92, 99, 100]. Undergoing a sestamibi scintigraphy study increases a patient's lifetime attributable risk for cancer incidence over baseline by 0.19% [92]. 4DCT exhibits a wide range of effective doses varying from 5.6 mSv to 28.5 mSv, with most studies reporting doses <15 mSv and two- and three-phase protocols conferring less radiation than four-phase protocols [45, 65, 92, 99–102]. Undergoing a 4DCT study increases a patient's lifetime attributable risk for cancer incidence over baseline by 0.52% [92]. Efforts to reduce the radiation dose from four-phase 4DCT protocols have spurred the use of two-phase protocols (consisting of precontrast and early arterial phases only) or even a single-phase protocol [103]. When used as a second-line modality, there is no significant difference in ability to lateralize parathyroid lesions between four-phase protocols and two- or three-phase protocols, including in reoperative patients and patients with multigland disease [63, 65]. Lastly, ^18^F-FCH PET/CT possesses an effective dose of 2.8 mSv [90]. Radiation doses exhibit a large range because protocols are highly specialized and vary from institution to institution. Nevertheless, the aforementioned imaging modalities are considered safe because the annual background radiation in the United States is 3 mSv and additional exposures of less than 15 mSv are considered low risk for carcinogenesis [99, 102].

## 13. Cost

Studies investigating the cost of preoperative imaging modalities are limited both in number and in the availability of cost data. A 2013 study examining Medicare payments and institutional charges calculated the costs of thin-cut 4DCT and sestamibi scintigraphy to be $1296 and $1112, respectively [99]. Meanwhile, the limited availability and high cost of PET radiotracers make PET unwarranted in uncomplicated or unexplored patients. Lubitz and colleagues concluded that ultrasound alone as a first-line modality followed by 4DCT in inconclusive cases is the most cost-effective strategy given the superior ability of 4DCT to enable minimally invasive parathyroidectomy [104]. Similarly, Wang and colleagues concluded that ultrasound and SPECT together as a first-line modality is the most cost-effective strategy, followed by 4DCT if the two initial studies are discordant [105]. The costs of imaging studies must be weighed against the benefits of highly sensitive preoperative localization, reductions in operating time, and increases in the likelihood of surgical cure.

## 14. Conclusion

The evaluation of persistent or recurrent disease in reoperative patients presents challenges for radiologists and surgeons alike. Accurate localization by preoperative imaging is a predictor of operative success. Inversely, negative or discordant preoperative imaging is a risk factor for operative failure, thus underscoring the importance of pursuing additional localization studies when first-line studies are inconclusive.

In this article, we review the common imaging modalities used to preoperatively localize parathyroid adenomas in the reoperative setting. We present the technique, accuracy, advantages, and disadvantages of each modality with respect to localizing persistent or recurrent disease. Unfortunately, there is no clear standard of care for the imaging localization of reoperative parathyroid pathology. Nevertheless, the present literature review enables us to recommend the following approach. Imaging in the reoperative setting should depend on which modalities were used during the patient's initial workup. First-line modalities in the reoperative setting should consist of both ultrasound and 4DCT, particularly in patients with rPHPT for whom evaluation of thyroid pathology is contributory to surgical management. Sestamibi scintigraphy is also an accurate initial modality in the reoperative setting. However, in practice, sestamibi scintigraphy is not repeated in patients with pPHPT or recent recurrence. Unfortunately, first-line imaging modalities are often negative or discordant in the reoperative setting, leaving many patients without curative options unless additional localization studies are pursued. Reoperative patients with negative or discordant first-line imaging should subsequently undergo PET/CT or conventional MRI before attempting invasive localization with SVS, which should be reserved for challenging cases in which several noninvasive modalities are inconclusive. When used in concert, the aforementioned techniques identify operative targets in challenging reoperative patients and thereby enable safe and curative parathyroidectomy.
